# A Jehovah's Witness with Acute Myeloid Leukemia Successfully Treated with an Epigenetic Drug, Azacitidine: A Clue for Development of Anti-AML Therapy Requiring Minimum Blood Transfusions

**DOI:** 10.1155/2014/141260

**Published:** 2014-10-02

**Authors:** Yumi Yamamoto, Akihito Kawashima, Eri Kashiwagi, Kiyoyuki Ogata

**Affiliations:** ^1^Metropolitan Research Center for Blood Disorders (MRC Japan), 1-45-46 Midorigaoka, Chofu, Tokyo 182-0001, Japan; ^2^Department of General Medicine, Shin-Yurigaoka General Hospital, Furusawa Tsuko 255, Asaoku, Kawasaki, Kanagawa 215-0026, Japan; ^3^Department of Hematology, Shin-Yurigaoka General Hospital, Furusawa Tsuko 255, Asaoku, Kawasaki, Kanagawa 215-0026, Japan

## Abstract

Therapy for acute leukemia in Jehovah's Witnesses patients is very challenging because of their refusal to accept blood transfusions, a fundamental supportive therapy for this disease. These patients are often denied treatment for fear of treatment-related death. We present the first Jehovah's Witness patient with acute myeloid leukemia (AML) treated successfully with azacitidine. After achieving complete remission (CR) with one course of azacitidine therapy, the patient received conventional postremission chemotherapy and remained in CR. In the case of patients who accept blood transfusions, there are reports indicating the treatment of AML patients with azacitidine. In these reports, azacitidine therapy was less toxic, including hematoxicity, compared with conventional chemotherapy. The CR rate in azacitidine-treated patients was inadequate; however, some characteristics could be useful in predicting azacitidine responders. The present case is useful for treating Jehovah's Witnesses patients with AML and provides a clue for anti-AML therapy requiring minimum blood transfusions.

## 1. Introduction

Therapy for acute leukemia patients who refuse blood transfusions, which is a cardinal supportive therapy during the induction period [1], is very challenging. These patients are often denied treatment for fear of treatment-related death. Among types of acute leukemia, acute myeloid leukemia (AML) excluding acute promyelocytic leukemia (APL) is the most challenging, whereas APL and acute lymphocytic leukemia (ALL) are, though very challenging, less risky because they are usually sensitive to relatively less myelosuppressive therapies, which are all-trans retinoic acid and combination of vincristine and glucocorticoid, respectively [2].

Azacitidine is a pyrimidine nucleoside analog of cytidine with antineoplastic activity [3]. Azacitidine is incorporated into DN2A, where it reversibly inhibits DNA methyltransferase, thereby blocking DNA methylation. Hypomethylation of DNA by azacitidine may activate tumor suppressor genes, which are often silenced by hypermethylation in tumor cells, resulting in an antitumor effect. Azacitidine is a therapeutic option for patients with myelodysplastic syndromes (MDS), who show cytopenia and often transform into AML. Azacytidine targets epigenetic changes in MDS and thereby presents fewer side effects compared with conventional chemotherapeutic drugs and is often applicable on an outpatient basis [4]. Recently, sporadic AML patients treated successfully with azacitidine have been reported [5].

In this paper, we present the first case of a Jehovah's Witness with AML who was successfully treated with azacitidine without blood transfusion. We also reviewed AML patients who accepted blood transfusions and were treated with azacitidine in the literature to provide a clue for the development of anti-AML therapy requiring minimum blood transfusions.

## 2. Case Report

The patient was a 33-year-old Japanese man, who developed dizziness one month ago. On admission, he was anemic and had low-grade fever (37.3°C). His blood cell count showed hemoglobin 7.3 g/dL, leukocytes 46.6 × 10^9^/L with 45.5% blasts, and platelets 29 × 10^9^/L. Bone marrow (BM) examination revealed hypercellular marrow with proliferation of myeloperoxidase-positive blasts (56.5% of all nuclear cells). Flow cytometry showed that blasts were positive for CD13, CD33, CD34, and HLA-DR and negative for lymphoid antigens. Chromosomal analysis of the BM cells showed normal karyotype. The diagnosis was “AML with maturation” according to the WHO 2008 classification. He was a Jehovah's Witness and did not accept any blood transfusions. Because he wished for the best treatment without transfusions, we initiated azacitidine therapy (75 mg/m^2^/day for 7 days, intravenous drip infusion) as an induction therapy on his fifth hospital day. In addition, he was given hydroxyurea (1500 mg/day) to control hyperleukocytosis from the 2nd to the 16th hospital day.

**Figure 1:**
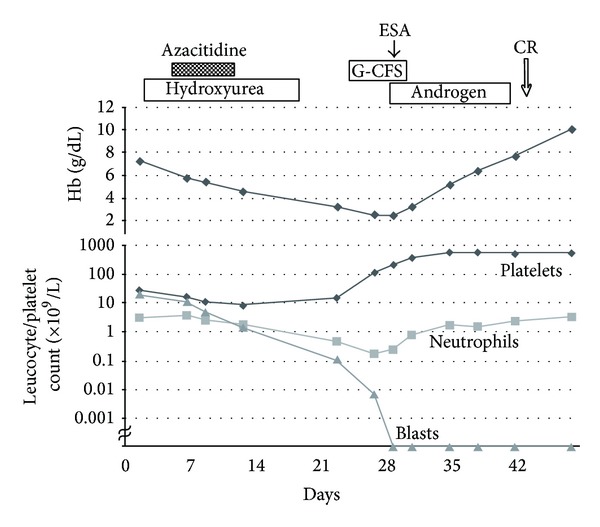
Clinical course of the patient during induction therapy with azacitidine. ESA: erythropoiesis-stimulating agent; G-CSF: granulocyte colony-stimulating factor.

The clinical course is summarized in Figure 1. The patient developed left axillary abscess on the 10th hospital day (day 10), which was treated with intravenous antibiotics and drainage with a small incision. During the induction therapy, his blood-cell nadir values were hemoglobin 2.5 g/dL, platelets 9 × 10^9^/L, and granulocytes 0.18 × 10^9^/L, and he experienced dizziness on walking, but bleeding tendency was mild and the axillary infection was well controlled. No other event occurred during the induction. The BM examination was carried out on day 20, which showed the disappearance of blasts and emergence of normal hematopoiesis. Granulocyte colony-stimulating factor (Filgrastim 75 *μ*g, subcutaneous injection on days 26–30), erythropoiesis-stimulating agent (darbepoetin alfa 120 *μ*g, subcutaneous injection on day 29), and an anabolic steroid (Primobolan 20 mg, oral administration on days 29–42) were used to enhance the recovery of normal hematopoiesis. The patient's blood cells recovered to the normal level, and the BM examination on day 43 confirmed complete remission. Flow cytometric analysis of BM cells showed no abnormal findings. The patient then received three cycles of conventional postremission chemotherapy (combination of idarubicin (12 mg/m^2^/day for 2 days) and enocitabine (a cytarabine-derivative, 300 mg/m^2^/day for 5 days)) and remained in remission with normal physical condition. He did not receive any blood transfusions.

## 3. Literature Review

Induction therapy for AML excluding APL in Jehovah's Witnesses patients has been reported in 25 cases [2, 15–28]. Patients in all cases received conventional chemotherapeutic drugs with either standard or reduced dose. Two of them also received gemtuzumab ozogamicin after determining that the chemotherapy was ineffective [20, 28]. Fifteen of these patients attained complete remission. The case presented in this paper is the 16th Jehovah's Witnesses patient with AML excluding APL who attained complete remission and the first Jehovah's Witnesses patient with AML who was treated with azacitidine.

Meanwhile, there are several reports in which AML patients were treated with azacitidine, supported with blood transfusion (Table 1) [5–14]. Because azacitidine is less toxic than conventional chemotherapy, a majority of these reports applied azacitidine therapy for patients who could not tolerate conventional chemotherapy. When newly diagnosed AML patients were treated with azacitidine, 10%–35% of patients attained complete remission, whereas chemorefractory and relapsed AML patients responded to a lower degree. Moreover, normal cytogenetics, low leukocyte count, and low blast percentages in the BM were associated with the response in newly diagnosed patients. The median time to response from the start of therapy was 2.5–4 months. In one report, transfusion requirement during induction therapy was examined and showed that the number of both erythrocyte and platelet transfusions was much lower in AML patients treated with azacitidine than in AML patients treated with conventional chemotherapy [5].

## 4. Discussion

AML is a fatal disease when untreated, characterized by the rapid growth of immature leukemic cells and a decrease in the number of mature normal blood cells. Induction chemotherapy is the first-line treatment of AML but is toxic for normal cells and requires the support of blood transfusion. Virtually all AML patients who have received standard induction chemotherapy have required multiple blood transfusions, including both erythrocyte and platelet products [1]. The main reason why most Jehovah's Witnesses patients with AML are denied treatment is a fear of treatment-related death because they do not accept blood transfusions. In this paper, we reported the first Jehovah's Witness patient with AML who was successfully treated with azacitidine. It has not shown that erythropoiesis-stimulating agent is effective for anemia in leukemia patients; however, we administered darbepoetin to the patient on day 29 because erythroid regeneration began in the bone marrow on day 20 but this regeneration did not improve anemia until day 29. It remains unclear whether darbepoetin played an important role in the improvement of anemia.

Because azacitidine is less toxic than conventional chemotherapy, several recent reports have indicated the treatment of AML patients who cannot tolerate conventional chemotherapy, mostly elderly patients [5–14]. In particular, one report showed that the number of blood transfusions required during the first course of induction chemotherapy is much lower in azacitidine-treated AML patients than in chemotherapy-treated AML patients [5]. If our patient received conventional chemotherapy, his blood-cell nadir values would be worse and lethal. Regarding CR rate, azacitidine is most likely inferior to conventional chemotherapy that achieves a CR rate of approximately 50% for elderly AML patients and 60%–80% for AML patients aged less than 60 years [29]. However, certain characteristics of AML, such as low leukocyte count, are associated with CR in azacitidine-treated AML. It is important to identify strong predictors of CR in azacitidine-treated AML patients, and, therefore, the analysis of a large number of azacitidine-treated AML patients, including those younger than 60 years old, is required. Moreover, augmenting the effect of azacitidine by other drugs, such as granulocyte colony-stimulating factor [30], and using other targeting drugs merit examination in future studies. Interestingly, in one report, AML patients were treated with either azacitidine or another demethylating agent, decitabine, and only decitabine induced complete remission [14].

## Figures and Tables

**Table 1:** Azacitidine therapy for AML in the literature.

Number of CR/number of all cases (CR rate)		Predictor for response^a^	Time to response^a^	Transfusions given during induction therapy period	Reference number
Newly diagnosed AML	Relapsed or refractory AML				
4/20 (20%)	—	Normal cytogenetics	Median 3 months (range 2–5 months)	No data	[6]
8/78 (10%)		No data	No data	No data	[7]
10/55 (18%)^b^	—	No data	No data	No data	[8]
2/20 (10%)^c^	0/20 (0%)	Lower BM blast percentages before therapy and day 15 of the therapy	Median 2.5 months (range 1–7 months)	No data	[9]
32/114 (28%)^d^	—	No data	No data	No data	[10]
8/35 (23%)	4/47 (8%)	Leukocyte count < 10 × 10^9^/L, newly diagnosed AML	No data	No data	[11]
9/26 (35%)	—	No data	Median 4 months (range 3–7 months)	RBC 2.7 times/month and platelets 0.3 times/month during the first 3 months^e^	[5]
15/155 (10%)		No data	Median 4 months	No data	[12]
13/55 (24%)	—	No data	Median 4 months (range 1–10 months)	No data	[13]
6/34 (18%)^f^	0/28 (0%)^f^	Newly diagnosed AML	Median 3.5 cycles of therapy	No data	[14]

^a^Response includes CR, partial remission, and hematological improvement defined in the previous report [6].

^b^Patients were AML with low bone marrow blast counts (blasts 20%–34%).

^c^Eight de novo AML and 12 AML transformed from MDS.

^d^Patients were treated with either azacitidine or decitabine and with or without histone deacetylase inhibitor.

^e^Transfusion requirement during the first course of induction chemotherapy was significantly less in patients treated with azacitidine compared with patients treated with conventional anti-AML chemotherapy (RBC transfusions: median 2.7 versus 7 times per month; platelet transfusions: median 0.3 versus 5 times per month).

^f^Patients were treated with either azacitidine or decitabine.
